# Development of an intervention to support the implementation of evidence-based strategies for optimising antibiotic prescribing in general practice

**DOI:** 10.1186/s43058-021-00209-7

**Published:** 2021-09-15

**Authors:** Aleksandra J. Borek, Anne Campbell, Elle Dent, Michael Moore, Christopher C. Butler, Alison Holmes, A. Sarah Walker, Monsey McLeod, Sarah Tonkin-Crine, on behalf of the STEP-UP study team, Philip E. Anyanwu, Aleksandra J. Borek, Nicole Bright, James Buchanan, Christopher C. Butler, Anne Campbell, Ceire Costelloe, Benedict Hayhoe, Alison Holmes, Susan Hopkins, Azeem Majeed, Monsey McLeod, Michael Moore, Liz Morrell, Koen B. Pouwels, Julie V. Robotham, Laurence S. J. Roope, Sarah Tonkin-Crine, Ann Sarah Walker, Sarah Wordsworth, Carla Wright, Sara Yadav, Anna Zalevski

**Affiliations:** 1 Nuffield Department of Primary Care Health Sciences, University of Oxford, Radcliffe Observatory Quarter, Woodstock Road, Oxford, OX2 6GG UK; 2 National Institute for Health Research (NIHR) Health Protection Research Unit in Healthcare Associated Infections and Antimicrobial Resistance, Imperial College London, London, UK; 3 Primary Care Population Sciences and Medical Education, Faculty of Medicine, University of Southampton, Southampton, UK; 4 NIHR Health Protection Research Unit in Healthcare Associated Infections and Antimicrobial Resistance, University of Oxford, Oxford, UK; 5 NIHR Oxford Biomedical Research Centre, Oxford, UK; 6 Nuffield Department of Medicine, University of Oxford, Oxford, UK; 7 Centre for Medication Safety and Service Quality, Pharmacy Department, Imperial College Healthcare NHS Trust, London, UK; 8 NIHR Imperial Patient Safety Translational Research Centre, Imperial College London, London, UK

**Keywords:** Implementation, Behaviour change, Qualitative, Antibiotic prescribing, Antimicrobial stewardship, Antibiotic resistance, Point-of-care C-reactive protein test, Delayed prescriptions, Communication, Intervention development

## Abstract

**Background:**

Trials show that antimicrobial stewardship (AMS) strategies, including communication skills training, point-of-care C-reactive protein testing (POC-CRPT) and delayed prescriptions, help optimise antibiotic prescribing and use in primary care. However, the use of these strategies in general practice is limited and inconsistent. We aimed to develop an intervention to enhance uptake and implementation of these strategies in primary care.

**Methods:**

We drew on the Person-Based Approach to develop an implementation intervention in two stages. (1) Planning and design: We defined the problem in behavioural terms drawing on existing literature and conducting primary qualitative research (nine focus groups) in high-prescribing general practices. We identified ‘guiding principles’ with intervention objectives and key features and developed logic models representing intended mechanisms of action. (2) Developing the intervention: We created prototype intervention materials and discussed and refined these with input from 13 health professionals and 14 citizens in two sets of design workshops. We further refined the intervention materials following think-aloud interviews with 22 health professionals.

**Results:**

Focus groups highlighted uncertainties about how strategies could be used. Health professionals in the workshops suggested having practice champions, brief summaries of each AMS strategy and evidence supporting the AMS strategies, and they and citizens gave examples of helpful communication strategies/phrases. Think-aloud interviews helped clarify and shorten the text and user journey of the intervention materials. The intervention comprised components to support practice-level implementation: antibiotic champions, practice meetings with slides provided, and an ‘implementation support’ website section, and components to support individual-level uptake: website sections on each AMS strategy (with evidence, instructions, links to electronic resources) and material resources (patient leaflets, POC-CRPT equipment, clinician handouts).

**Conclusions:**

We used a systematic, user-focussed process of developing a behavioural intervention, illustrating how it can be used in an implementation context. This resulted in a multicomponent intervention to facilitate practice-wide implementation of evidence-based strategies which now requires implementing and evaluating. Focusing on supporting the uptake and implementation of evidence-based strategies to optimise antibiotic use in general practice is critical to further support appropriate antibiotic use and mitigate antimicrobial resistance.

**Supplementary Information:**

The online version contains supplementary material available at 10.1186/s43058-021-00209-7.

Contributions to the literature

- This paper reports a systematic process to developing digital behavioural interventions, drawing on the Person-Based Approach and combining theoretical modelling with qualitative research with target users.
- It illustrates the use of this approach in an implementation context and the value of involving target users at all stages of intervention development and planning implementation.
- It shows that professionals valued a brief, multicomponent implementation intervention with online training, physical resources, champions and practice meetings.
- This study adds knowledge on how to develop implementation interventions for clinical settings and how to best engage clinicians as the target users.

## Background

Antimicrobial resistance (AMR) poses a severe global threat to public health and modern medicine. Without effective antimicrobial medicines, many common infections and routine medical and dental procedures will become life-threatening. One of the main contributing factors to AMR is over-use of antibiotics, especially in primary care where most antibiotics are prescribed [1]. Many antimicrobial stewardship (AMS) strategies have been used to optimise antibiotic prescribing and reduce antibiotic use, especially for acute respiratory infections in primary care [2–4]. Some have been implemented nationally or regionally in England as part of the Quality Premium incentive scheme, e.g. antibiotic prescribing targets; monitoring, feedback and benchmarking of antibiotic prescribing rates; and audit and feedback to individual prescribers by prescribing advisors from Clinical Commissioning Groups (CCGs) [1, 5–8]. Among many others, AMS strategies include using enhanced communication skills and patient leaflets, point-of-care C-reactive protein testing (POC-CRPT) and delayed/back-up antibiotic prescriptions (DPs). These strategies have been tested in clinical trials and systematic reviews of trials support their effectiveness in reducing antibiotic prescribing/use in primary care [2, 4, 9].

The GRACE-INTRO trial [10–12], a large international study (including England and Wales), involved developing and testing two strategies to reduce antibiotic prescribing for lower respiratory tract infections: (i) online training for general practitioners (GPs) in enhanced communication skills, supported by interactive use of a patient booklet (‘Caring for Coughs’), and (ii) using POC-CRPT. The 2x2 factorial trial tested each intervention alone, and both combined, against usual care. Participating practices were also asked to appoint a lead GP to organise a structured meeting on prescribing issues. The trial showed that both strategies were effective in reducing antibiotic prescriptions, with the combined arm showing the biggest effect [10]. At a 12-month follow-up, the online communication skills training showed a longer-lasting effect [11]. Other trials in England have also shown interactive use of leaflets can help support communication when not prescribing antibiotics for children (‘When Should I Worry’ booklet [13]) and adults with respiratory infections (Infosheets [14]). UK-based trials also have shown that giving patients DPs (instead of immediate antibiotic prescriptions) with a good explanation is an effective strategy to safely reduce antibiotic use by patients: showing that only 33–39% of patients use antibiotics when given a DP and that the strategy helps prevent complications and reduce re-consultations and future consultations for similar illnesses [15–17].

While trials show that these three AMS strategies are safe and effective in reducing antibiotic prescriptions/use, their uptake in the ‘real world’ in English general practices is unknown and, anecdotally, limited and inconsistent. The GRACE-INTRO training and booklet are currently not publically available, although similar (‘STAR: Stemming the Tide of Antibiotic Resistance’) communication training is [18, 19]. The ‘When Should I Worry’ booklet is available online [20], but it is unclear how widely it is used. POC-CRPT is not routinely available in English general practices, except a few local pilots [21–23]. Finally, DPs are and can be used, but clinicians have varied views and approaches to DPs, many report not using DPs at all, and not coding them consistently [24–26]. Therefore, addressing this gap between trial evidence and real-world implementation of evidence-based AMS strategies is an important step following development and testing of interventions. It may be particularly important for those practices that remain high prescribers of antibiotics despite the availability of AMS strategies. These practices may require additional support to implement such strategies (e.g. the three aforementioned evidence-based but under-utilised AMS strategies) to help them optimise antibiotic prescriptions/use.

This study aimed to develop and evaluate an intervention to support the implementation (henceforth ‘implementation intervention’ or ‘intervention’) of three evidence-based AMS strategies (communication skills training and use of patient leaflets, POC-CRPT, and DPs) in high antibiotic prescribing general practices in England. It is a part of a larger research programme called STEP-UP (‘Improving the uptake and SusTainability of Effective interventions to promote Prudent antibiotic Use in Primary care’) [27]. This paper describes the intervention development process and provides a comprehensive description of the implementation intervention.

## Methods and results

### Overview of methods

In our research, we drew on elements of the Person-Based Approach (PBA) [28–30] which is a systematic approach to developing behaviour change interventions, particularly those with digital components. It is distinctive from other approaches in its focus on involving people from the target user populations through qualitative research and co-design. The PBA helps integrate evidence and theory-based intervention development (focussing on psychosocial and behavioural change processes and techniques) with a user-centred design that improves the usability, acceptability and engagement of technology-based interventions. It has been used to develop a wide range of health-related behaviour change interventions to target patients and healthcare professionals [31], including the GRACE-INTRO intervention [10, 32] and a digital intervention to reduce antibiotic prescriptions in hospitals [33].

The main elements of the PBA are as follows: (i) undertaking qualitative research with people from the target user populations at all stages of intervention development (starting with exploring psychosocial and contextual influences on the target behaviour) and (ii) developing ‘guiding principles’ (comprising design objectives and key intervention features to achieve objectives) and theoretical integration that shows how the intervention will address the target behaviour and determinants. Table 1 summarises the steps taken in our intervention development. Progress through the steps was iterative so later steps fed back into earlier steps (e.g. feedback from design workshops influenced the theoretical modelling). In this paper, we describe the two stages of planning and developing the intervention; the implementation and evaluation of the intervention will be reported separately.

**Table 1:** Summary of the implementation intervention development process

Stages	Steps	Person-Based Approach [28–30]
Planning & design of the intervention	1. Defining the problem in behavioural terms, identifying target behaviours, users and influences on behaviour (literature scoping, qualitative research, expert input) 2. Creating guiding principles & theoretical modelling (logic modelling)	Intervention planning: • Literature scoping and review • Qualitative research with target users • Formulating guiding principles (intervention design objectives and key features of intervention) • Behavioural analysis and construction of logic model
Developing the intervention (components)	3. Developing (drafting) intervention components & materials (design workshops) 4. Refining intervention materials (think-aloud interviews)	Intervention optimisation: • Draft/refine intervention materials • Qualitative piloting of draft materials • Refine guiding principles • Revisit behavioural analysis and refine logic model
Implementing and evaluating the intervention	5. Implementing the intervention in real-life context 6. Mixed-methods evaluation	Mixed-methods process evaluation: • Quantitative research • Qualitative research with users • Triangulation • Examine theory-based questions drawn from logic model

### Implementation intervention planning and design

#### Step 1: Defining the problem, target behaviours, users and influences

##### Methods

In the initial part of the intervention development process, we drew on the expertise of the research team and existing literature and conducted primary qualitative research. The research team were a multidisciplinary group (GPs, a pharmacist, a psychologist, sociologists, statisticians and health economists and health service researchers), including researchers experienced in optimising antibiotic prescribing in primary care. We used the team’s expertise throughout the intervention development but especially in the initial planning.

As part of a related study [34, 35], we conducted two scoping reviews of (i) studies of AMS strategies (interventions) and (ii) qualitative studies on influences on antibiotic prescribing; both included healthcare professionals in UK primary care and focussed on prescribing for acute respiratory infections. We used this evidence to identify evidence-based AMS strategies and modifiable influences on antibiotic prescribing.

Given that much existing qualitative research with healthcare professionals about AMS strategies was within trials and unspecific to implementation or high-prescribing practices, we conducted our own qualitative research. The methods of this focus group study are reported in detail elsewhere [26]. In brief, we held nine focus groups with 50 professionals (3–11 per practice) in high-prescribing practices (i.e. top 20% for antibiotic prescribing based on 2017 PrescQIPP data [6]) in England to better understand practice professionals’ views on antibiotic prescribing, optimisation and implementing/using POC-CRPT and DPs. The focus groups were conducted by AJB and AC using a semi-structured topic guide and lasted 49–87 min. Practices were reimbursed for participation. The data were analysed using an inductive thematic approach in NVivo software by four researchers (AJB, AC, STC, ED), and analytic saturation was achieved. The findings informed our choice of targeted influences and potential intervention components.

##### Results

The problem and target behaviours: Drawing on the research team’s expertise and experience, we identified the problem as low uptake and inconsistent use of evidence-based AMS strategies in English general practice. Thus, the target behaviour was use of evidence-based AMS strategies in general practice consultations for acute infections. We hypothesised that increased use of AMS strategies would decrease prescribing of (immediate) antibiotics. Using evidence from systematic reviews and clinical trials of AMS strategies in England, expertise of the research team, consideration of whether support already existed for an AMS strategy, and whether the support was within scope of and feasible in our study, we selected three AMS strategies: (i) communication skills training with interactive use of patient leaflets, (ii) POC-CRPT and (iii) DPs. For communication skills training and POC-CRPT, we aimed to support implementation of the training and resources developed and tested in the GRACE-INTRO study [10–12, 32]. For DPs, existing trials did not target clinician behaviour but rather aimed to assess the impact of DPs on patient behaviour (whether they used antibiotics when given a DP), patient satisfaction, likelihood of re-consulting for the same or different illness and the safety of delaying antibiotics [15, 16]; thus, we aimed to develop materials targeted at clinicians to promote DP use. Other effective AMS strategies exist that were not included [2–4], e.g. monitoring, feedback and benchmarking (peer comparison) of antibiotic prescribing rates, and audit and feedback to individual prescribers, have already been implemented in England [7, 8]; electronic clinical decision support tools/systems (which may involve different features and would require remote changes to and integration into different clinical systems software [14]) and patient education were considered unfeasible within and outside of scope of our study.

Target users: Although antibiotic prescribing in general practices has reduced in recent years, studies show that a proportion of general practices remain high-prescribing [36–38]. Therefore, we identified the ‘users’ or ‘population’ to target by our intervention as healthcare professionals in high antibiotic prescribing practices (i.e. in the top quarter of antibiotic prescribing in England). We targeted prescribers and non-prescribers in these practices because communication skills (with leaflets) and POC-CRPT can be used by both prescribers and non-prescribers, whereas DPs are used by prescribers. However, we also envisaged that implementation of the strategies in practices may involve non-clinical practice professionals who support clinicians (e.g. receptionists triaging patients for POC-CRPT or managing DPs to be collected later). Therefore, we agreed that our intervention would target all general practice professionals, with a primary focus on prescribers.

Influences on antibiotic prescribing: We fully report the identified influences on antibiotic prescribing and optimisation in our review of qualitative studies in the UK [34, 35], our focus group study in high-prescribing practices [26] and in Supplementary File 1. From these, we selected influences considered important, modifiable by an intervention and most feasible to address. The iterative nature of the development process meant these targeted influences were further refined, particularly following the workshops (step 3). Table 2 shows which influences were targeted in the intervention and by which components. Supplementary File 2 reports the targeted influences matched with the Theoretical Domains Framework categories [39].

**Table 2:** Influences on antibiotic prescribing and optimisation

Types of influences	Influences on antibiotic prescribing & optimisation (identified and fully reported in [26, 34, 35])	Intervention components
Evidence & education	1. Clinician awareness of evidence & guidelines 2. Peer discussion & learning 3. Clinician training/education on antibiotic prescribing 4. Advice from & influence of relevant experts	Website Practice meetings, champion Website Website
Clinical experience & confidence	5. Clinical experience & confidence	Website, training
Clinical assessment	6. Clinical uncertainty about illness aetiology, severity and/or progression 7. Additional diagnostic information from testing	POC-CRPT POC-CRPT
Perceptions of patient’s expectations & satisfaction	8. Perceptions of patient expectations of antibiotics 9. Preserving a good relationship with patient, patient satisfaction & avoiding conflict	3 AMS strategies 3 AMS strategies
Communication skills & strategies	10. Ability to elicit & manage patient’s concerns & expectations 11. Ability to reassure & safety-net 12. Perceived importance of shared decision making 13. Ability & motivation to educate patients in consultations	Comms 3 AMS strategies Comms, DP Website
Time & workload	14. Time pressure & workload (e.g. wanting to save time & prevent future consultations) 15. Consultation length (& not wanting to lengthen consultations)	Website Website
Professional role & ethos	16. Perceptions of professional role & ethos	Website, champion
Awareness & perceptions of responsibility for AMS	17. Clinician awareness/knowledge of & attitude to AMS	Champion
Monitoring, feedback & accountability	18. Receiving feedback on prescribing	Practice meeting
Perceptions of own & others’ prescribing	19. (In)Consistent approach to antibiotic prescribing between clinicians/organisations	Practice meeting, champion
Attitudes to & use of AMS strategies*	20. Views on & use of strategies 21. Access to resources to use strategies	3 AMS strategies 3 AMS strategies, resources
Additional influences identified in the focus groups in relation to POC-CRPT and DP [26]	22. Perceived fit of strategies with clinical roles and experience 23. Perceived usefulness of strategies as social tools to negotiate treatment and educate patients 24. Ambiguities about strategies (incl. evidence, when and how to use them, impact on antibiotic prescribing/use) 25. Practice context (incl. ease of access, availability of dispensary, deprivation, patient characteristics, time pressures, costs, logistics/workflows)	Website 3 AMS strategies Website, practice meeting Practice meeting, champions, resources

*Comms* communication skills training (including interactive use of leaflets), *DP* delayed antibiotic prescriptions, *POC-CRPT* point-of-care C-reactive protein testing

*Strategies identified in the qualitative studies (in usual care, outside of trials) included only DPs and leaflets, and not communication skills training or POC-CRPT; however, it can be assumed that similar influences are relevant to all three AMS strategies

#### Step 2: Creating guiding principles and theoretical modelling

##### Methods

After identifying target behaviours and influences, we established guiding principles for the intervention. These incorporated design objectives for the intervention and its key features (i.e. how it would address these objectives).

We developed two logic models to illustrate the intended change mechanisms. The first described individual-level processes of how the AMS strategies facilitate change in clinicians’ antibiotic prescribing behaviour. The second described practice-level processes of how the implementation intervention was intended to facilitate change in practice-wide implementation and clinicians’ use of the AMS strategies. The logic models were refined throughout intervention development. We also identified formats by which to deliver the intervention (i.e. intervention components).

##### Results

Table 3 summarises the guiding principles for the implementation intervention. We identified the importance of the intervention fitting the local context; thus, rather than developing a generic, prescriptive implementation plan, we aimed to support autonomy and tailoring by encouraging practices to develop their own implementation plan. The intervention provided professionals with a choice of AMS strategies to use, and how, by offering a range of resources, including multiple patient leaflets (printed and electronic), and two types of POC-CRPT equipment (qualitative and quantitative, which could be stored differently). The feedback from the design workshops (step 3) and think-aloud interviews (step 4) stressed the importance of intervention materials being concise and user-friendly, due to demands on professionals’ time, and the importance of the intervention coming from a trustworthy source.

**Table 3:** Guiding principles for the implementation intervention

Design objectives	Key features of the implementation intervention
To support practice-wide implementation and use of the AMS strategies	• Promote use of the three evidence-based AMS strategies in general practices • Intervention features aimed at all practice professionals to support both individual and practice-level change • Support practices to develop and agree practice-wide, consistent approaches to using the AMS strategies • Nominate practice champions to provide peer encouragement and support
To support autonomy and enable tailoring in how the AMS strategies are used	• Offer a choice of leaflets and POC-CRPT equipment • Non-prescriptive on how practices should implement strategies • Non-prescriptive on how clinicians should use strategies (including clinical situations)
To persuade users that information and AMS strategies are evidence-based and trustworthy	• Clear references to evidence and guidelines • Endorsed by the President of the Royal College of General Practitioners • Videos and testimonials of practising clinicians explaining how they use the strategies • Description of intervention as developed by a multidisciplinary university-based team (including practising clinicians), with non-commercial research funding
To be brief and concise	• Website to take less than an hour to read • Text as concise as possible • Use of expandable boxes on the website with additional details • Handouts for clinicians with key messages maximum of one A4 page
To be easy to use and navigate	• Similar structure of webpages for each strategy • Access to all sections of the website from the navigation bar (no need to go through the website sequentially, but sequential use possible)

**Fig. 1:**
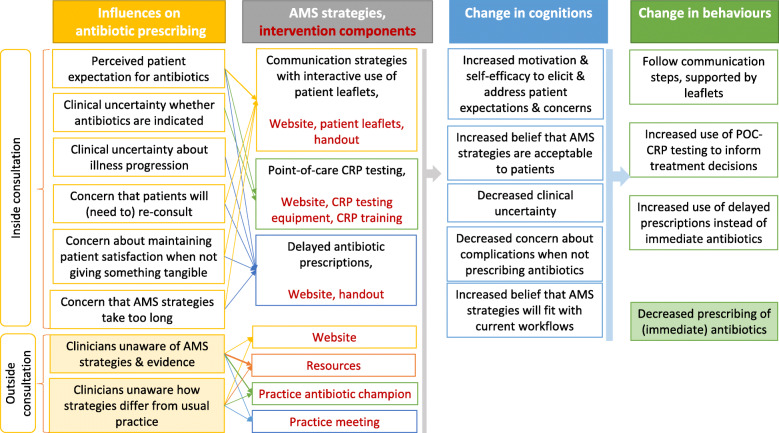
Logic model 1 for the three AMS strategies

Our first logic model (Fig. 1) illustrates how the three AMS strategies are hypothesised to influence individual-level change in antibiotic prescribing behaviour. We identified the key target influences on antibiotic prescribing: clinicians’ perceptions of patient expectations for antibiotics (influences 8 and 23, Table 2), addressed by all three AMS strategies; clinical uncertainty about indication for antibiotics and illness severity and progression (influence 6), addressed by POC-CRPT and DPs; concern that patients will (need to) re-consult (influence 14) and/or will be dissatisfied if not receiving something tangible (e.g. prescription, leaflet) (influence 9), addressed by communication strategies and DPs; and concern that the AMS strategies take too long or would lengthen consultations (influence 15), addressed by the information about communication strategies and DPs provided on the website as part of the intervention. Figure 1 shows these influences were addressed by the three AMS strategies directly and/or by the components of the implementation intervention and then were hypothesised to facilitate change in clinicians’ cognitions, leading to higher uptake of the three AMS strategies and, consequently, decreased prescribing of (immediate) antibiotics.

**Fig. 2:**
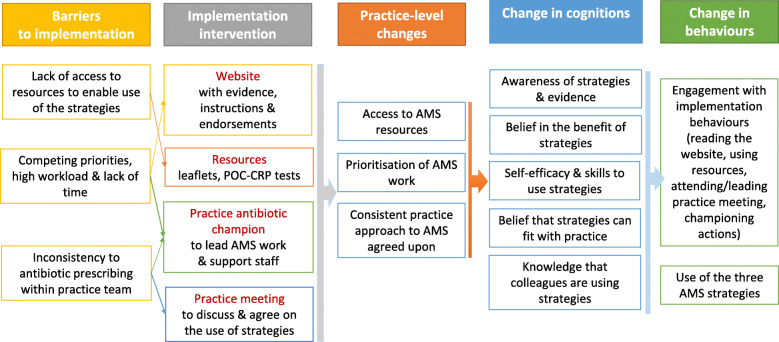
Logic model 2 for the implementation intervention

The second logic model (Fig. 2) illustrates how the implementation intervention was hypothesised to facilitate the practice-level implementation of the three AMS strategies. In particular, we identified the lack of access to resources to enable use of these strategies (influence 21, Table 2) as a critical barrier, addressed by providing printed leaflets/booklets and POC-CRPT equipment. Competing priorities, with high workloads and insufficient time (influence 14), constituted also key barriers to prioritising antibiotic optimisation and implementation of new strategies in practices and were addressed by identifying practice antibiotic champions to lead AMS and support colleagues in using AMS strategies. Finally, perceived inconsistency between clinicians’ antibiotic prescribing and use of AMS strategies (influence 19) was a barrier due to concern about patients’ expectations for antibiotics, dissatisfaction or re-consultations if not prescribed antibiotics; this was addressed through the champion and practice meetings that aimed to ensure more consistent, practice-wide approach.

### Implementation intervention development and refinement

#### Step 3: Developing intervention components and materials (design workshops)

##### Methods

We conducted four workshops to discuss intervention components: two in March and two in June 2019, with one workshop with professionals and one with citizens (i.e. members of the public) at each time-point. Workshops were in-person and lasted approximately 3 h each. Informed consent was taken from all participants at the start, and participants were reimbursed for participation. All workshops were audio-recorded and transcribed verbatim.

Health professionals included GPs, nurses and CCG professionals responsible for AMS in primary care. For the first workshop, we invited participants from our earlier focus-group study, through professional networks, and representatives from local CCGs. These invitations were sent by email. For the citizen workshop, we advertised through a website promoting opportunities for public involvement in NHS, public health and social care research (www.peopleinresearch.org). All attendees of the first workshops were invited to the second workshops and we sought new participants as needed.

The first professional workshop aimed to gather views and feedback on the three AMS strategies and implementation support. We facilitated discussions to allow participants to voice their views and experiences around key questions. The first citizens’ workshop focused on ‘talking about infections and antibiotics with your GP’ and ‘helping GPs discuss back-up/delayed prescriptions with patients’. We presented citizen participants with hypothetical scenarios (e.g. consulting a GP with a sore throat and a GP using certain communication strategies) to prompt discussions. Professionals and citizens were given handouts with the key questions and scenarios to enable them to add comments if they wished. Two researchers in each workshop made field notes.

After the first set of workshops, we combined field notes with data from the transcripts, participants’ comments from the handouts, and relevant suggestions from the focus group study. All suggestions were summarised and discussed by the study team and used to develop draft implementation intervention materials. For the website, we developed a website design brief and worked with a professional web designer. We drafted content for the webpages and developed prototypes of the webpages and resources.

The second professionals’ workshop aimed to discuss and collect feedback on the content, design and delivery of the intervention components. We presented draft webpages on the three AMS strategies and resources. The second citizens’ workshop further explored discussing back-up/delayed prescriptions, as well as views/suggestions on helping other types of prescribers to discuss prescribing decisions, and on different types of patient leaflets. Following the second set of workshops, we compiled the comments and suggestions as before and agreed changes to be made.

##### Results

The first set of workshops were attended by 11 professionals (five GPs, five CCG pharmacists/prescribing advisors, one practice nurse prescriber) and by 14 citizens. The second set of workshops were attended by nine professionals (five GPs, three CCG pharmacists/advisors, one nurse prescriber) and by 10 citizens.

Following the first set of workshops, we made decisions about the intervention components (e.g. website sections, having practice rather than CCG champions). We made changes to the content of the training on communication strategies and communication about DPs (e.g. included examples of helpful/unhelpful communication strategies) (see Table 4).

**Table 4:** Summary of feedback from the first set of design workshops and resulting changes

Main comments & suggestions	Main changes to the intervention
*Communication skills –professional workshop:* • Already use communication skills but are open to new ideas for things to say to patients (especially those perceived as ‘difficult’ to communicate with about antibiotics); need to highlight what is new; suggestions to call it ‘enhancing your communication skills’, ‘finding the right words’ or ‘tips/ideas for things to say to patients’. • Provide example phrases but keep short to avoid lengthening the consultation. • Leaflets should be discussed with patients, not just handed-out. *Communication skills – citizen workshop:* • Provide examples of helpful and unhelpful communication strategies (e.g. need for acknowledging illness, addressing pain, discussing side effects of antibiotics). • Leaflets can be helpful but should not replace the conversation.	• Changed the communication webpage name & title of the handout for clinicians. • Highlighted that strategies may be particularly helpful with patients who are expecting antibiotics. • Example phrases provided on website. • More emphasis on side effects of antibiotics and using leaflets interactively.
*POC-CRPT – professional workshop only*: • Need to be clear that tests shouldn’t be done in all patients; practices need an agreed protocol for when and how they will use the tests, and complete training in using and interpreting the tests. • Tests perceived as potentially helpful with ‘borderline’ patients and to benchmark clinicians’ ‘gut feeling’.	• Additional training to be offered by a provider/trainer. • Suggestions of when tests can help included on webpage.
*Delayed prescriptions (DP) – professional workshop:* • Calling them ‘back-up’ prescriptions preferred as more reassuring than ‘delayed’. • DP can be confusing to patients (‘why are you giving a prescription when explaining that antibiotics aren’t needed?’). • The 6R model for communicating about DP should be combined with the CHESTSSS communication steps; clinicians are unlikely to explicitly go through a list of 6Rs. Training should be simpler and shorter. • Post-dating prescriptions can be seen as patronising and lead to patient complaints. • Need for a consistent approach to DP across prescribers. *Delayed prescriptions (DP) – citizen workshop:* • ‘Back-up’ preferred to ‘delayed’, or explanation that the prescription is ‘in case’. • DP perceived as confusing (‘why offer it after explaining that antibiotics won’t help?’); patients would prefer to re-consult rather than have a DP. • Need something to help patients remember how/when to use the DP. • Need clear communication on when antibiotics will work or not, and on when to use the DP (‘if you’re getting worse’ is too vague). • The 6R model perceived as long; suggestions to shorten it to a more meaningful acronym (e.g. WAIT). • Post-dating would be perceived as insulting as suggesting clinician’s lack of trust in the patient.	• Used ‘back-up/delayed’ wording throughout website/resources. • Clarified the suggested use of DP with prognostic uncertainty rather than when patients don’t need antibiotics to avoid mixed messages; examples phrases provided to avoid confusion. • Removed 6Rs and replaced with acronym WAIT to refer to elements of communication about DP. • DP linked to communication strategies (CHESTSSS) on website. • Examples of helpful and unhelpful explanations of DP added to website.
*Champions – professional workshop only:* • Champions for antibiotics/AMS are helpful, otherwise focus is lost among other priorities. The champion needs to be someone who is already part of practice team (e.g. not someone from CCG). • Need to involve the whole practice team and agree a consistent approach to antibiotics. • Practice antibiotic champions could lead practice meetings/training.	• Focus on practice champions rather than CCG champions. • Practice meetings to involve discussions and promote consensus on practice-wide approaches.
*Other key comments about implementation intervention – professional workshop only:* • Need to help clinicians see how the training will be useful for them and their practice. • Make the training a part of the existing electronic system/training programme. • Present information in varied ways to cater for different preferences and learning styles. • Keep the training/information as brief as possible; use bullet points rather than long sentences or paragraphs. • Have summary sheets (up to one side of A4) to briefly summarise/highlight key messages.	• Refer to benefits of using the strategies promoted to optimise antibiotics on the website home page. • Made the text more concise; used more bullet points, boxes, and tables. • Provided two handouts for clinicians to summarise communication and DP.

The second set of workshops led to further changes to the website and resources (Table 5). As a result of the discussions, we presented the three AMS strategies in a purposeful order: (1) communication skills and leaflets (most sustainable, universal and cheapest); (2) POC-CRPT (potentially helping to reduce inappropriate prescriptions but more costly and time-consuming); and (3) DP (helping to reduce immediate antibiotic prescriptions). We included videos available from other interventions for communication strategies and DPs and provided links to instructional videos on using the POC-CRPT analyser. Workshop participants expressed different views on whether the website should be offered as online training to be completed sequentially with a certificate of completion, or if it should be used flexibly—with any section/webpage accessible directly (non-sequentially). Following our guiding principles to support choice, autonomy and tailoring, we decided to enable flexible use. This meant users could access webpages directly from the menus; however, we also included links to sequential webpages at the bottom of each page. Professionals also wanted clear instructions on when to use and not use POC-CRPT (e.g. which patients/conditions). This was an important clinical question and so we provided links to existing guidelines and evidence for when to use POC-CRPT. Finally, professionals also discussed that the role of practice champions may need to be incentivised. To help provide an intrinsic incentive, we explained the importance of this role on the website—we were unable and considered it impractical to offer any extrinsic incentives (e.g. financial).

**Table 5:** Main changes to the intervention following the second set of design workshops

Website section	Main changes made in result of the suggestions in the second workshop
Overall website	• Replaced references to ‘GPs’ with ‘prescribers’. • Added a Resources webpage with a list of all downloadable leaflets and resources, links to additional external resources (e.g. TARGET toolkit) and research papers/evidence. • Reduced the number of separate webpages for each section and moved non-essential text into expandable boxes for use if people want more details.
Home webpage	• Main focus on the three AMS strategies, with short explanations what they are and direct links to these sections. • Presented the three AMS strategies in a purposeful order; communication skills and leaflets, POC-CRPT and DP.
Section on communication skills and leaflets	• Added videos with a GP giving examples of communication strategies (helpful phrases). • Clarified that despite focus on acute infections, these strategies can be applicable to other types of consultations. • Shortened the text; highlighted examples found particularly helpful and novel by workshops participants. • Added sections on ‘benefits of leaflets’ and ‘how to use leaflets to engage patients’.
Section on POC-CRPT	• Addressed the concern that POC-CRPT may increase demand and appointments for tests. • Clarified when to use and not use POC-CRPT.
Section on DP	• Addressed the concern that patients use DPs immediately by referring to trial evidence that shows that most (2/3) patients don’t end up using DP. • Clarified that DP should not be offered if the GP doesn’t think antibiotics are clinically needed, but rather instead of immediate antibiotics. • Highlighted the potential benefits of DP (e.g. reducing re-consultations or ‘doctor-shopping’).
Section on Implementation Support and Champions	• Explained who is meant by a practice Antibiotic Champion. • Explained why champions are important. (Suggestion to offer financial incentives was unaddressed as unfeasible.) • Suggested that champions may identify another professional to help with some activities.

#### Step 4: Refining intervention materials (think-aloud interviews)

##### Methods

Think-aloud interviews with health professionals were used to collect detailed feedback to refine the online component of the intervention and resources. Professionals were recruited from those involved in previous stages of the research (e.g. workshops) and through research team networks. Interviews (lasting about an hour) took place remotely or in person. All participants gave informed consent and were reimbursed for their time.

Interviews were conducted by AJB, AC, and ED between July and October 2019. Participants were given a link and asked to freely navigate and read the website during the interview. They were asked to read the webpages commenting (‘thinking aloud’) about the content, design, navigation and any other aspects if they wished to. Interviews were audio-recorded and detailed notes were made during the interviews.

Each participant’s suggestions were inserted into a table and then assessed using pre-existing criteria for deciding whether to make modifications and MoSCoW ranking (i.e. Must, Should, Could, Would like to change, or no change) [40]. Changes that were deemed ‘Must do’ or simple to do were addressed immediately after the interviews; other changes were addressed after every few interviews. We continued the interviews until no major suggestions for changes were made and data saturation was reached.

##### Results

Twenty-two professionals completed think-aloud interviews (12 GPs, 4 practice nurse prescribers, 2 CCG prescribing advisors, 2 practice pharmacy prescribers, 1 pharmacy prescriber, and 1 advanced paramedic practitioner). The interviews lasted 37–73 (mean 56) minutes. Thirteen were conducted by telephone, six face-to-face, and three by Skype.

Table 6 presents examples of suggestions and how we addressed them. The main changes were made to the layout of webpages, improved navigation, further condensing and reducing text and providing links directly to guidelines and evidence. The most mixed views related to whom the website would be useful (some found it useful, others suggested it would be useful to less experienced prescribers), preferences and views on each of the three strategies, perceived lack of incentive to read the website, and whether it should be formatted like an instructional course with a certificate of completion. The most positive views related to content are as follows: examples of communication strategies and what not to say when explaining DPs (to avoid mixed messages to patients), information on typical duration of common infections, instructions on using the POC-CRPT equipment and interpreting test results and suggestions for champions to address common questions and concerns. Participants also liked references to guidelines and evidence, and institutional logos and endorsements were perceived as adding credibility. After many changes, in later interviews, they also reported the text as clear, concise and ‘punchy’.

**Table 6:** Summary of feedback from think-aloud interviews and resulting changes

Website section	Example suggestions from think-aloud interviews with health professionals	How they were addressed
Home page	• The website was perceived as unattractive without pictures. • Unclear why these three AMS strategies are promoted. • Unclear logo. More ‘branding’ would seem helpful.	➢ Added pictures for each AMS strategy. ➢ Clarified reasons for promoting the three strategies. ➢ Unchanged as participants held different views and was not considered a priority.
Section on communication strategies	• Example phrases and mock conversations liked. • Perceived difficulty with using leaflets with patients where English isn’t their first language; unclear how they could access leaflets in other languages from the website. • Some disliked the mnemonic CHESTSSS, seen as hard to remember. • Too many webpages to go through to access the leaflets.	➢ Added more example phrases. ➢ Highlighted availability of leaflets in other languages and provided a link to them. ➢ CHESTSSS retained as covering all key elements. ➢ Moved all information on leaflets to one webpage.
Section on POC-CRPT	• References to NICE guideline should be highlighted. • Would like more information on using and interpreting results for different conditions. • Add information about manufacturer’s training and quality control tests. • Questioned if the website/project was funded by CRP test producers. • Would like a template or Standard Operating Procedures (SOP) for using POC-CRPT in practices.	➢ NICE guidelines and trial evidence highlighted. ➢ No evidence for different conditions (other than respiratory infections) so no change. ➢ Added details on training and quality control. ➢ Clarified sources of funding. ➢ No template/SOP provided; suggested questions to agree on practice approach in meeting slides.
Section on DP	• Concerned about ‘red flags’ and need for reconsultation or urgent care. • Highlight the information on typical duration of common infections. • Some confusion about the different names for DP used.	➢ Added information on red flags (e.g. sepsis). ➢ Added a specific table on typical duration of infections. ➢ ‘Back-up/delayed’ retained as different people prefer/use different names.
Section on Implementation Support and Champions	• More focus on addressing concerns and suggested actions for champions, rather than reasons for promoting AMS. • Practice meeting slides need to be shorter (for 5–10-min meetings). • Unclear who the resources for antibiotic champion webpage/link is for. • Information seemed targeted at those who already are champions and not encouraging people to become one.	➢ Shortened the text on reasons and benefits, and expanded actions for champions. ➢ Shortened slides and divided into multiple sets. ➢ Called the webpage ‘implementation support’. ➢ Edited text to clarify the information is for everyone promoting prudent prescribing.

### Antibiotic Optimisation implementation intervention

Here we describe the final version of the implementation intervention. Behaviour change techniques [41] that were included in the intervention are reported in Supplementary File 2. Further website details are in Supplementary File 3. The completed Template for Intervention Description and Replication (TIDieR) checklist [42] is in Supplementary File 4.

**Fig. 3:**
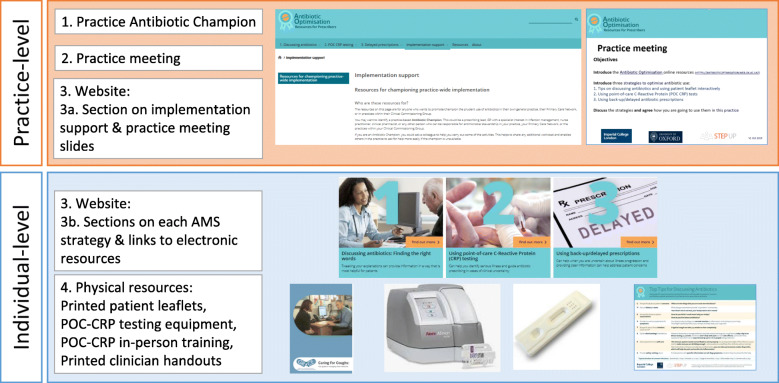
Components of the Antibiotic Optimisation implementation intervention

The implementation intervention has four components (Fig. 3), described below. As depicted in the logic models, the intervention targeted changes at practice-level and individual-level.

First, practices are asked to identify a practice-based antibiotic champion to lead implementation of the AMS strategies in the practice and to support and encourage other clinicians. The antibiotic champion could be a prescribing lead, GP or nurse practitioner interested in infections and antibiotic optimisation, or any other clinician responsible for AMS in the practice. The role could also be shared by two professionals.

Second, practices are asked to organise at least one practice meeting focused on antibiotic optimisation and the AMS strategies. We suggest meeting(s) is/are led by the antibiotic champion(s). The first meeting aims to raise awareness and motivation to optimise antibiotics: it should focus on introducing the three AMS strategies, the antibiotic optimisation website and associated resources and facilitate discussions and agreement on when and how the strategies are implemented in the practice. Subsequent meetings (every 2–3 months) are suggested to help remind prescribers about the strategies and resources, review implementation and inform new or locum staff about them.

Component 3a, targeted at practice-level change, is the ‘Implementation Support’ section on the Antibiotic Optimisation website. This introduces the champion role, suggests actions for champions to promote implementation of the AMS strategies and helps address common questions and concerns. It includes guidance to help champions lead introductory and subsequent meetings and four sets of PowerPoint meeting slides designed to take 5–10 min to go through—one set providing an overview of the resources and strategies and one set each for the three strategies.

Component 3b, targeted at individual clinicians, are three AMS strategies (communication strategies and patient leaflets, POC-CRPT, DPs) sections on the Antibiotic Optimisation website. Each section includes evidence-based instructions and rationale on how and why to use the strategies, examples, evidence and guidelines, and videos or quotes from clinicians describing how and why they use the strategies. There are also links for electronic patient leaflets and handouts for clinicians (i.e. short reminder sheets with top tips for discussing antibiotics, interpreting POC-CRPT results, recording POC-CRPT results as part of training and discussing and coding DPs).

The Antibiotic Optimisation website is a key component of the intervention. It is primarily targeted at prescribers, but can be used by any professional involved in implementing the three AMS strategies (e.g. practice nurses doing POC-CRPT). It can be used flexibly, e.g. non-sequentially as any section and page can be accessed directly or sequentially by links at the bottom of each page. All professionals have access to all parts of the website. Our think-aloud interviews indicated that reading the whole website takes up to 1 h. Supplementary File 3 reports the content of each section.

The fourth component provides resources to enable use of the AMS strategies. These include printed versions of patient leaflets/booklets and clinician handouts, and two types of POC-CRPT equipment. In our focus groups, time was reported to be a critical factor and participants considered one of the three tests discussed to be too long for general practice consultations so we excluded it from the intervention. The POC-CRPT website section directs users to providers of the POC-CRPT equipment who offer in-person training. We also suggest a training task: all prescribers use the POC-CRPT on the first 10 patients with acute cough and record the results on a handout.

The next step of this study involved implementing the implementation intervention in high-prescribing general practices in England and a mixed-methods evaluation. Following this, we are in the process of incorporating the resources into existing, publicly available AMS resources. Until made publicly available, the website and resources can be provided from the authors on reasonable request. There is no specific number of times or period over which the intervention should be delivered; rather, we envisage that health professionals engage with it in ways that suit them and when they want additional support with implementing the three AMS strategies.

## Discussion

In this paper, we describe the process of developing the Antibiotic Optimisation intervention to promote and support the uptake and implementation of three evidence-based AMS strategies in high-prescribing general practices. This was an iterative process of intervention planning, design, development and refinement, in which we combined evidence, theoretical modelling and qualitative research with target users and stakeholders.

The Antibiotic Optimisation implementation intervention was targeted at health professionals in general practice. While we focused on the context of general practice and involved primary care stakeholders, the final intervention has some similarities with the Antibiotic Review Kit (ARK) intervention to safely review and reduce antibiotic prescriptions in hospitals [33]. Both have components targeted at individuals (e.g. online tool/website, patient leaflets) and at teams—‘implementation teams’ in ARK and practice teams in our intervention (e.g. implementation guidance/website, champions). Implementation requires both individual and organisational change, so the targets for, and processes in, implementation interventions are more complex and multi-level than interventions focussed on individuals only. Other studies also evaluated the implementation of intervention components similar to our implementation intervention (e.g. online training, champions, outreach visits, leaflets) [43–45]. However, unlike in these studies, we distinguished AMS strategies (e.g. POC-CRPT, DPs) that aim to influence antibiotic prescribing decisions from implementation strategies (e.g. champions, website) that aim to influence the uptake and implementation of the AMS strategies in practices. This is illustrated by our two logic models where we specified the intended ‘mechanisms of action’ of different types of intervention components.

Digital components (websites, e-learning modules) are important in our and many other interventions and are commonly used to provide training and education for clinicians. A systematic review of eight trials in primary care found that digital education on antibiotic management was more effective in improving knowledge and likely more cost-effective than traditional education [46]. Online AMS training for all patient-facing staff was also one of the highest-ranked AMS interventions by primary care stakeholders in previous research [47].

Nevertheless, engagement with digital interventions remains challenging. Health professionals in the ARK study were sceptical about digital education due to high workloads and limited time (the 30-min ARK e-module was shortened to 10 min) [33]. Similarly, we found a main barrier to optimising antibiotics and engaging with AMS strategies in general practice was limited time. Thus, we revised the website, handouts and practice meeting slides until they were as concise as possible, but it remains uncertain how acceptable the time required to engage with them is. We also decided not to offer the 10-min POC-CRPT as it was deemed too time-consuming by clinicians. In our focus groups [26], we found that an important barrier to using POC-CRPT and DPs was ambiguity about evidence and when, and how, to use the strategies; and professionals in our workshops and think-aloud interviews asked for evidence and clear guidelines on using these strategies. Consequently, we needed to strike a balance between making the intervention short and not losing important content, and between providing evidence and guidance while allowing flexibility and autonomy.

We have previously identified the importance of national and local champions as facilitators to engagement and implementation in our qualitative research with CCG and general practice professionals [7] and with primary care stakeholders [47]. Growing literature on champions and leaders in primary care supports their important role in facilitating implementation of AMS strategies [43, 48] and other initiatives (e.g. [49, 50]). However, a qualitative study with Norwegian GPs showed a need for leadership training and tensions between GPs’ clinical and leadership roles [51]. We initially explored involving CCG prescribing advisors as champions but professionals in our workshops suggested practice-based champions more suited to help implement the strategies within practice contexts and support colleagues. However, they also suggested providing incentives (e.g. paying for their time), which was unfeasible in our study and complex in the real-world context. Finally, as we previously found [7], in-person communication in practices was preferred (e.g. practice meetings), although challenging with time constraints. Wider, national implementation of interventions often means that digital, remote delivery is more feasible without the in-person components (helping to lower cost and time requirements). For example, the ‘STAR’ communication training initially involved digital training and a practice-based seminar, but it is currently available online only [18, 19]. The ‘TARGET’ (‘Treat Antibiotics Responsibly, Guidance, Education, Tools’) training initially also had digital and in-person elements, and its national implementation involves training trainers to continue delivering in-person training [52, 53]. Our intervention promotes practice meetings led by practice-based professionals, making it potentially more flexible and sustainable in real-world settings, enabling ownership of the initiatives and implementation, and consistent practice between professionals.

A recent framework for planning, conducting and disseminating AMR intervention research has called for research to be more responsive to stakeholder needs and for interventions to be better designed, including consideration of behavioural determinants, theory and logic-models [54]. Different approaches and tools have been established and used to develop health-related behaviour change interventions [55]; e.g. Medical Research Council guidance [56], Intervention Mapping [57], the Behaviour Change Wheel [58] and the Person-Based Approach (PBA) [28–30]. These approaches can be also used to develop interventions to support implementation. We drew on the PBA for its suitability for designing interventions with digital components and focus on stakeholder engagement and co-design with target users; thus, helping increase the likelihood of the intervention being relevant, acceptable and feasible.

However, we found challenges with the PBA. For example, it encourages a digital delivery early in the intervention development process, which may not always be the most optimal delivery format. Moreover, in complex behaviours, such as implementation of (also complex) AMS strategies, it is challenging to identify the most important influences on behaviour and determinants of change. For example, we identified over 41 types of influences on antibiotic prescribing from qualitative research. It was unclear which were most important and what other unidentified influences (e.g. subconscious) may also be important. We tried to address influences that were commonly reported and that resonated most with stakeholders and the study team. This resulted in trying to address many influences but to different extents (e.g. some only by including brief information on the website). Finally, it is unclear how the many approaches to behaviour change intervention development [55] may be incorporated with the many implementation frameworks and models which exist [59]. In our research, we were aware of the concepts included in the implementation frameworks but did not use them explicitly; an implementation framework will be used to guide the evaluation in the implementation study.

### Strengths and limitations

We followed a pre-defined, systematic process to developing the intervention, identifying and addressing views and experiences of target users, while also incorporating evidence- and theory-based elements. We engaged a relatively large and diverse number of relevant stakeholders. We also engaged citizens (members of the public) to better understand and incorporate patient perspectives. In the qualitative sub-studies, we reached data saturation. A strength was also our multidisciplinary team of experienced researchers and clinicians, who led and advised on the intervention development. We followed guidance for reporting interventions [42] and intervention development studies [60] (checklists are in Supplementary Files 4 and 5).

Limitations of the study, and thus potentially of the developed intervention, remain. We acknowledge that there are other effective AMS strategies that could be considered for implementation (e.g. clinical decision support tools) and that our focus on the three evidence-based AMS strategies was to some extent influenced by the expertise and interests of the research team and the scope of our study. The interviewers were involved in intervention development so there was potential for socially desirable answers in the workshops and think-aloud interviews. However, our data show that participants expressed critical comments. Professionals attending the workshops were likely to be more interested in AMS and optimising antibiotics so their views and experiences might have differed from professionals less engaged in AMS. However, we also incorporated findings and suggestions from focus groups conducted in high-prescribing practices. Some professionals involved had previous experience of implementing/using the AMS strategies and could share their experiences, whereas others had not used some of the AMS strategies (e.g. POC-CRPT) which meant that they approached the strategies with fresh eyes. We conducted think-aloud interviews only and did not conduct interviews after giving people time to use the website/resources on their own (as suggested by Bradbury et al. [61]); these will be part of the mixed-methods evaluation in the next stage. Not all suggestions were feasible or practical to address and there are wider contextual influences that affect high antibiotic prescribing [62], which are beyond the target for one practice-based intervention. In our qualitative data collection, we relied on participants’ reports of views and experiences. These may differ from actual behaviour and do not uncover subconscious influences. Thus, other methods, such as observations, may be also needed (e.g. in future studies). Finally, it is as yet unclear how applicable and fitting the intervention has been during, and will be after, the COVID-19 pandemic which has, at least temporarily, transformed how general practices work.

## Conclusions

In this article, we report the development of an implementation intervention which followed a systematic, user- and stakeholder-focussed process. We describe the multicomponent ‘Antibiotic Optimisation’ intervention that aims to promote the implementation of evidence-based AMS strategies in general practices. Our intervention targets individual- and practice-level behaviour change processes. In the next stage of our research, the intervention has been piloted and evaluated in an implementation study. With increasing numbers of AMS strategies and interventions and growing trial-based evidence of effectiveness, it is now critical to work towards bridging the gap between evidence and practice and improve implementation of evidence-based strategies, particularly in high-prescribing practices that need to further optimise antibiotic prescribing.

## Supplementary Information

** Supplementary File 1.** Complete table of influences on antibiotic prescribing and optimisation. **Supplementary File 2.** Theoretical Domains Framework constructs and Behaviour Change Techniques. **Supplementary File 3.** Details of the Antibiotic Optimisation implementation intervention. **Supplementary File 4.** The TIDieR checklist. **Supplementary File 5.** The GUIDED checklist.

## Data Availability

The datasets analysed during this study are available from the corresponding author on reasonable request.

## Abbreviations

AMR: Antimicrobial resistance
AMS: Antimicrobial stewardship
CCG: Clinical Commissioning Group
DPs: Delayed/back-up prescriptions
GP: General practitioner
PBA: Person-Based Approach
POC-CRPT: Point-of-care C-reactive protein testing
