# Supplementary material for: Development of an intervention to support the implementation of evidence-based strategies for optimising antibiotic prescribing in general practice
Source: Implement Sci Commun. 2021 Sep 15;2:104. doi: 10.1186/s43058-021-00209-7 (PMC8441243; doi:10.1186/s43058-021-00209-7)
Supplement: Supplementary file 1 — Complete table of influences on antibiotic prescribing and optimisation. Supplementary File 2. Theoretical Domains Framework constructs and Behaviour Change Techniques. Supplementary File 3. Details of the Antibiotic Optimisation implementation intervention. Supplementary File 4. The TIDieR checklist. Supplementary File 5. The GUIDED checklist. [file 43058_2021_209_MOESM1_ESM.pdf]

**Supplementary Files**

**For**

**"Development of an intervention to support the implementation of evidence-based strategies for optimising antibiotic prescribing in general practice"**

## Contents

Supplementary File 1. Complete table of influences on antibiotic prescribing and optimisation ........ 2

Supplementary File 2. Theoretical Domains Framework constructs and Behaviour Change Techniques ........ 4

Supplementary File 3. Details of the Antibiotic Optimisation implementation intervention ... 8

Supplementary File 4. The TIDieR checklist ........ 10

Supplementary File 5. The GUIDED checklist ........ 13

References ........ 14

## Supplementary File 1. Complete table of influences on antibiotic prescribing and optimisation

**Table S1. Influences on antibiotic prescribing and optimisation**

| Types of influences | Influences on antibiotic prescribing & optimisation (identified and reported in (1,2)) | Intervention components |
|---|---|---|
| **Evidence & education** | 1. Clinician awareness of evidence & guidelines | Website |
| | 2. Peer discussion & learning | Practice meetings, champion |
| | 3. Clinician training/education on antibiotic prescribing | Website |
| | 4. Advice from & influence of relevant experts | Website |
| **Clinical experience & confidence** | 5. Clinical experience & confidence | Website, training |
| | 6. Experience of and concern about adverse events resulting from prescribing decisions | Not addressed |
| | 7. GP's preference for certain antibiotics | Not addressed |
| **Clinical assessment** | 8. Clinical assessment of signs & symptoms & making a diagnosis | Not addressed |
| | 9. Clinical uncertainty about illness aetiology, severity and/or progression | POC-CRPT |
| | 10. Patient's risk of complications or poor outcomes | Not addressed |
| | 11. Patient's perception & presentation of illness | Not addressed |
| | 12. Access to patient's medical records or history | Not addressed |
| | 13. 'Gut feeling' (intuition) about patient & illness | Not addressed |
| | 14. Additional diagnostic information from testing | POC-CRPT |
| **Knowledge & perceptions of the patient** | 15. Prior knowledge of & familiarity with the patient | Not addressed |
| | 16. Perceptions of the patient | Not addressed |
| | 17. Ability to re-assess or follow-up the patient | Not addressed |
| | 18. Patient's social factors | Not addressed |
| **Perceptions of patient's expectations & satisfaction** | 19. Perceptions of patient expectations of antibiotics | 3 AMS strategies |
| | 20. Preserving a good relationship with patient, patient satisfaction & avoiding conflict | 3 AMS strategies |
| | 21. Patient's preference for certain antibiotics | Not addressed |
| **Communication skills & strategies** | 22. Ability to elicit & manage patient's concerns & expectations | Comms |
| | 23. Ability to reassure & safety-net | 3 AMS strategies |
| | 24. Perceived importance of shared decision making | Comms, DP |
| | 25. Ability & motivation to educate patients in consultations | Website |
| **Time & workload** | 26. Timing of consultation, access to medical services | Not addressed |
| | 27. Time pressure & workload (e.g., wanting to save time & prevent future consultations) | Website |
| | 28. Consultation length (& not wanting to lengthen consultations) | Website |
| **Professional role & ethos** | 29. Perceptions of professional role & ethos | Website, champion |
| **Awareness & perceptions of** | 30. Prioritising immediate pressures vs. long-term consequences of inappropriate prescribing | Not addressed |

| | | |
|---|---|---|
| **responsibility for AMS** | 31. Clinician awareness/knowledge of & attitude to AMS | Champion |
| **Monitoring, feedback & accountability** | 32. Use of monitoring & audit<br>33. Receiving feedback on prescribing<br>34. Accountability for own prescribing (or its lack) | Not addressed<br>Practice meeting<br>Not addressed |
| **Perceptions of own & others' prescribing** | 35. Perceptions of own prescribing as compared to others<br>36. (In)Consistent approach to antibiotic prescribing between clinicians/organisations | Not addressed<br>Practice meeting, champion |
| **Costs associated with prescribing** | 37. Perception of costs related to antibiotic prescribing | Not addressed |
| **Legal issues** | 38. Concern with legal issues (or patient complaints) resulting from not prescribing antibiotics | Not addressed |
| **Attitudes to & use of AMS strategies*** | 39. Views on & use of strategies<br>40. Access to resources to use strategies<br>41. Use of financial incentives | 3 AMS strategies<br>3 AMS strategies, resources<br>Not addressed |
| **Additional influences identified in the focus groups in relation to POC-CRPT and DP (3)** | 42. Perceived fit of strategies with clinical roles and experience<br>43. Perceived usefulness of strategies as social tools to negotiate treatment and educate patients<br>44. Ambiguities about strategies (incl. evidence, when and how to use them, impact on antibiotic prescribing/use)<br>45. Practice context (incl. ease of access, availability of dispensary, deprivation, patient characteristics, time pressures, costs, logistics / workflows) | Website<br>3 AMS strategies<br>Website, practice meeting<br>Practice meeting, champions, resources |

*Abbreviations used in the table: Comms – communication skills training (including interactive use of leaflets), DP – delayed antibiotic prescriptions, POC-CRPT – point-of-care C-reactive protein testing.*

** Strategies identified in the qualitative studies (in usual care, outside of trials) included only DPs and leaflets, and not communication skills training or POC-CRPT; however, it can be assumed that similar influences are relevant to all three AMS strategies.*

## Supplementary File 2. Theoretical Domains Framework constructs and Behaviour Change Techniques

Table S1 contains the influences on antibiotic prescribing and optimisation addressed by the Antibiotic Optimisation implementation intervention and the three antimicrobial stewardship strategies. All influences were categorised using the Theoretical Domains Framework (TDF) constructs (4) and are fully reported elsewhere (1,2). Behaviour change techniques (BCTs) relevant to corresponding influences and intervention components were added subsequently using the BCT taxonomy version 1 (5).

**Table S2. Influences on antibiotic prescribing, Theoretical Domains Framework (TDF), intervention components and Behaviour Change Techniques (BCTs)**

| Types of influences | Influences on antibiotic prescribing & optimisation (1,2) | TDF constructs | Intervention components (examples) | BCTs |
|---|---|---|---|---|
| **Evidence & education** | 1. Clinician awareness of evidence & guidelines | Knowledge | Website (sections with evidence for using each strategy) | • Instruction on how to perform the behaviour<br>• Social support (practical)<br>• Credible source<br>• Information about health consequences |
| | 2. Peer discussion & learning | Social influences | Practice meetings, champions | |
| | 3. Clinician training/education on antibiotic prescribing | Knowledge | Website (sections on evidence & using each strategy) | |
| | 4. Advice from & influence of relevant experts | Social influences | Website (videos & endorsements of GPs, credible study team) | |
| **Clinical experience & confidence** | 5. Clinical experience & confidence | Beliefs about capabilities | Website (instructions & videos on using the strategies)<br>Training (in using POC-CRPT) | • Instruction on how to perform the behaviour<br>• Behavioural practice/rehearsal<br>• Demonstration of the behaviour |
| **Clinical assessment** | 6. Clinical uncertainty about illness aetiology, severity and/or progression | Beliefs about consequences | POC-CRPT | • Adding objects to the environment<br>• Information about health consequences |

| | | | | |
|---|---|---|---|---|
| | 7. Additional diagnostic information from testing | Environmental context & resources | POC-CRPT | |
| **Perceptions of patient's expectations & satisfaction** | 8. Perceptions of patient expectations for antibiotics | Social influences | 3 AMS strategies | • Information about health consequences<br>• Information about social & environmental consequences<br>• Instruction on how to perform the behaviour<br>• Demonstration of the behaviour<br>• Information about others' approval<br>• Behaviour substitution<br>• Prompts/cues |
| | 9. Preserving a good relationship with patient, patient satisfaction & avoiding conflict | Intentions, skills | 3 AMS strategies | |
| **Communica-tion skills & strategies** | 10. Ability to elicit & manage patient's concerns & expectations | Skills, social influences | Comms, leaflets, handout | • Instruction on how to perform the behaviour<br>• Information about social & environmental consequences<br>• Prompts/cues<br>• Demonstration of the behaviour<br>• Information about others' approval<br>• Behaviour substitution |
| | 11. Ability to reassure & safety-net | Skills | 3 AMS strategies, leaflets, handouts | |
| | 12. Perceived importance of shared decision making | Social influences, social/ professional role & identity | Comms, DP | |
| | 13. Ability & motivation to educate patients in consultations | Skills, intentions | Website (instructions on how to use the strategies), handouts | |
| **Time & workload** | 14. Time pressure & workload (e.g., wanting to save time & prevent future consultations) | Environmental context & resources, beliefs about consequences | Website (reassurance about time to use the strategies) | • Information about social & environmental consequences<br>• Demonstration of the behaviour |

| | | | | |
|---|---|---|---|---|
| | 15. Consultation length (& not wanting to lengthen consultations) | Environmental context & resources | Website (reassurance about time to use the strategies) | |
| **Professional role & ethos** | 16. Perceptions of professional role & ethos | Social/ professional role & identity | Website (appeal to health professionals' role in providing good quality care), Champions | • Information about social & environmental consequences<br>• Valued self-identity<br>• Discrepancy between current behaviour and goal<br>• Social support (practical)<br>• Commitment |
| **Awareness & perceptions of responsibility for AMS** | 17. Clinician awareness/ knowledge of & attitude to AMS | Knowledge, beliefs about consequences | Champions | • Information about health consequences<br>• Information about social & environmental consequences<br>• Discrepancy between current behaviour and goal<br>• Social support (practical) |
| **Monitoring, feedback & accountability** | 18. Receiving feedback on prescribing | Knowledge, social influence | Practice meetings | • Feedback on behaviour<br>• Discrepancy between current behaviour and goal<br>• Commitment<br>• Credible source |
| **Perceptions of own & others' prescribing** | 19. (In)Consistent approach to antibiotic prescribing between clinicians/ organisations | Social influences, environmental context & resources | Practice meetings, champions | • Restructuring the social environment<br>• Social support (practical)<br>• Commitment<br>• Information about others' approval |
| **Attitudes to & use of AMS strategies*** | 20. Views on & use of strategies | Beliefs about consequences, intentions | 3 AMS strategies | • Information about health consequences<br>• Information about social & environmental consequences |

| | | | | |
|---|---|---|---|---|
| | 21. Access to resources to use strategies | Beliefs about consequences, intentions | 3 AMS strategies - resources (POC-CRPT equipment, leaflets) | • Adding objects to the environment |
| **Additional influences identified in the focus groups in relation to POC-CRPT and DP (3)** | 22. Perceived fit of strategies with clinical roles & experience | Social/ professional role & identity | Website (instruction on how to use the strategies within clinical roles and experience) | • Information about health consequences<br>• Information about social & environmental consequences<br>• Instruction on how to perform the behaviour<br>• Adding objects to the environment<br>• Discrepancy between current behaviour and goal<br>• Information about others' approval<br>• Demonstration of the behaviour<br>• Commitment |
| | 23. Perceived usefulness of strategies as social tools to negotiate treatment and educate patients | Beliefs about consequences | 3 AMS strategies<br>Website (instructions on how to communicate) | |
| | 24. Ambiguities about strategies (incl. evidence, when and how to use them, impact on antibiotic prescribing/use) | Knowledge, skills | Practice meetings, champions (encouraged discussion of the strategies and how to use them) | |
| | 25. Practice context (incl. ease of access, availability of dispensary, deprivation, patient characteristics, time pressures, costs, logistics / workflows) | Environmental context & resources | Practice meetings, champions (encouraged to tailor the use of strategies in the practice context),<br>Resources (POC-CRP tests, leaflets) | |

*Abbreviations used in the table: BCTs – behaviour change techniques, Comms – communication skills training (including interactive use of leaflets), DP – delayed antibiotic prescriptions, POC-CRPT – point-of-care C-reactive protein testing, TDF – Theoretical Domains Framework.*

** Strategies identified in the qualitative studies (in usual care, outside of trials) included only DPs and leaflets, and not communication skills training or POC-CRPT; however, it can be assumed that similar influences are relevant to all three AMS strategies.*

## Supplementary File 3. Details of the Antibiotic Optimisation implementation intervention

**Table S3. Content of the Antibiotic Optimisation website**

| Website section | Content |
|---|---|
| **Home page** | • Introductory sentence and an endorsement from the President of the Royal College of General Practitioners<br>• Short descriptions and links to sections on the three AMS strategies<br>• Benefits of optimising antibiotic prescribing |
| **Discussing antibiotics (finding the right words)** | • Enhancing your discussions with patients (introduction)<br>• Downloadable (pdf) summary sheet 'Top Tips for Discussing Antibiotics'<br>• Explanation and examples of the communications strategies following the CHESTSSS acronym:<br>  o C – Ask specifically about patient's CONCERNS<br>  o H – Discuss History and exam<br>  o E – Ask specifically about patient EXPECTATIONS<br>  o S – Provide non-serious explanation for SYMPTOMS<br>  o T – Be specific about illness TIMELINE / usual course<br>  o S – Explain SHORTCOMINGS of antibiotics<br>  o S – Advise patients how to SELF-CARE<br>  o S – Provide SAFETY-NETTING advice<br>• Short videos of a GP discussing and using this approach<br>• Page on evidence for using this approach (how it can help, evidence of benefits, common questions and concerns)<br>• Page on discussing a leaflet interactive in consultations (why and how to use leaflets interactively)<br>• Downloadable (pdf) copies of leaflets with information about them:<br>  o 'Treating Your Infections' leaflets for adults (Public Health England, https://www.rcgp.org.uk/clinical-and-research/resources/toolkits/amr/target-antibiotics-toolkit/leaflets-to-share-with-patients.aspx)<br>  o 'Caring for Coughs' booklet for adults (from the GRACE-INTRO trial (6))<br>  o 'When Should I Worry?' booklet for parents/carers of children with respiratory infections (7)<br>  o Infosheets for adults and children with respiratory infections (8) |
| **Using point-of-care CRP testing** | • Introduction to POC-CRP testing and to the two types of tests provided as part of the intervention/study<br>• Downloadable summary sheets for interpreting the POC-CRP test results<br>• Training task to carry out POC-CRP tests with 10 patients presenting with acute cough and recording the CRP results on the downloadable template sheet provided<br>• Using each type of the POC-CRP tests (instructions on how to use the equipment, links to instructional videos, how to interpret the results, practicalities, support from the manufacturer, information on test performance)<br>• Why use POC-CRP tests (how they can help you, evidence of benefits, common questions and concerns, quotes from prescribers who have used POC-CRP tests in their practices)<br>• When to use POC-CRP tests (which patients, considering risk factors) |

| | |
|---|---|
| **Using back-up/ delayed prescriptions** | • How to explain back-up/delayed prescriptions to patients, details on:<br>  o Reasons for giving it<br>  o Specific number of days to wait<br>• Examples of how to discuss DPs (what some prescribers might say, how some patients might interpret this, and alternative suggestions)<br>• Five ways to issue DPs<br>• Coding DPs with READ/SNOMED codes<br>• Downloadable (pdf) reminder sheet for clinicians about how to discuss and code DPs<br>• Why use back-up/delayed prescriptions (how they can help, evidence of benefits, common questions and concerns)<br>• Videos of a GP discussing the evidence related to using DPs |
| **Implementation support** | • Resources for championing practice-wide implementation (who are they for and what is their purpose)<br>• Addressing common questions and concerns about the three AMS strategies<br>• Leading practice meetings and meeting slides<br>• Other actions to champion the three AMS strategies |
| **Resources** | • Printable support materials (summary sheets and instructions for clinicians)<br>• Patient leaflets / booklets<br>• External AMS resources<br>• Websites to check antibiotic prescribing rates in practices<br>• References to supporting evidence |
| **About** | • Information about the STEP-UP study<br>• Information on funding for the study<br>• Contact details |

## Supplementary File 4. The TIDieR checklist

**Table S4. The TIDieR checklist (Template for Intervention Description and Replication)** (9)

| Item number | Item | Where located | |
|---|---|---|---|
| | | Manuscript (page or appendix number) | Other (details) |
| | **BRIEF NAME** | | |
| **1.** | Provide the name or a phrase that describes the intervention. | p. 14 | Antibiotic Optimisation implementation intervention |
| | **WHY** | | |
| **2.** | Describe any rationale, theory, or goal of the elements essential to the intervention. | p. 5, Table 3 | ______________ |
| | **WHAT** | | |
| **3.** | Materials: Describe any physical or informational materials used in the intervention, including those provided to participants or used in intervention delivery or in training of intervention providers. Provide information on where the materials can be accessed (e.g. online appendix, URL). | pp. 15-16 | ______________ |
| **4.** | Procedures: Describe each of the procedures, activities, and/or processes used in the intervention, including any enabling or support activities. | pp. 15-16 | ______________ |
| | **WHO PROVIDED** | | |
| **5.** | For each category of intervention provider (e.g. psychologist, nursing assistant), describe their expertise, background and any specific training given. | n/a | Intervention was mostly provided online. Optional training in using POC-CRPT equipment was offered by trainers from the commercial provider, but not part of the intervention. |

| | | | |
|---|---|---|---|
| | **HOW** | | |
| **6.** | Describe the modes of delivery (e.g. face-to-face or by some other mechanism, such as internet or telephone) of the intervention and whether it was provided individually or in a group. | pp. 15-16 | Intervention is delivered to practices online, and physical resources are delivered to practices by post/couriers. |
| | **WHERE** | | |
| **7.** | Describe the type(s) of location(s) where the intervention occurred, including any necessary infrastructure or relevant features. | n/a | Intervention will be delivered in general practices. |
| | **WHEN and HOW MUCH** | | |
| **8.** | Describe the number of times the intervention was delivered and over what period of time including the number of sessions, their schedule, and their duration, intensity or dose. | n/a | Implementation and evaluation will be reported separately. |
| | **TAILORING** | | |
| **9.** | If the intervention was planned to be personalised, titrated or adapted, then describe what, why, when, and how. | p. 9, Table 3 | ______________ |
| | **MODIFICATIONS** | | |
| **10.**[‡] | If the intervention was modified during the course of the study, describe the changes (what, why, when, and how). | n/a | Actual intervention delivery is not reported in this paper which only reports the intervention development process. |
| | **HOW WELL** | | |
| **11.** | Planned: If intervention adherence or fidelity was assessed, describe how and by whom, and if any strategies were used to maintain or improve fidelity, describe them. | n/a | As above. |

| | | | |
|---|---|---|---|
| **12.**[‡] | Actual: If intervention adherence or fidelity was assessed, describe the extent to which the intervention was delivered as planned. | n/a | As above. |

## Supplementary File 5. The GUIDED checklist

**Table S5. The GUIDED checklist (guideline for reporting intervention development studies)** (10)

| **Item** | **Page in manuscript** | **Other** |
|---|---|---|
| 1. Report the context for which the intervention was developed. | 5 | |
| 2. Report the purpose of the intervention development process. | 5 | |
| 3. Report the target population for the intervention development process. | 8 | |
| 4. Report how any published intervention development approach contributed to the development process. | 6, Table 1 | |
| 5. Report how evidence from different sources informed the intervention development process. | 5, 7-8, 9, 12-13, 14 | I.e. Results sections for each step |
| 6. Report how/if published theory informed the intervention development process. | 9-10, Figures 1 and 2 | I.e. Programme theory (guiding principles and logic models) |
| 7. Report any use of components from an existing intervention in the current intervention development process. | 5 | |
| 8. Report any guiding principles, people or factors that were prioritised when making decisions during the intervention development process. | 9-10, Table 3 | |
| 9. Report how stakeholders contributed to the intervention development process. | 11-12, 13, Tables 4-6 | I.e. Steps 3 and 4 |
| 10. Report how the intervention changed in content and format from the start of the intervention development process. | 12-14 | I.e. We describe changes made to the intervention in result of involving stakeholders/target users throughout |
| 11. Report any changes to interventions required or likely to be required for subgroups. | n/a | The intervention was designed for general practice teams with different components targeted at sub-groups (e.g., champions). This is described in results sections and description of the intervention. |
| 12. Report important uncertainties at the end of the intervention development process. | 17, 18 | |
| 13. Follow TIDieR guidance when describing the developed intervention. | 14, Supplementary File 4 | |
| 14. Report the intervention development process in an open access format. | yes | We plan to publish the article open access. |

## References


1. Borek AJ, Wanat M, Atkins L, Sallis A, Ashiru-Oredope D, Beech E, et al. Optimising antimicrobial stewardship interventions in English primary care: a behavioural analysis of qualitative and intervention studies. BMJ Open. 2020 Dec 1;10(12):e039284.

2. Borek AJ, Wanat M, Roberts N, Atkins L, Sallis A, Tonkin-Crine S. Exploring the Implementation of Interventions to Reduce Antibiotic Use (ENACT) Study: Report. London: Public Health England; 2019.

3. Borek AJ, Campbell A, Dent E, Butler CC, Holmes A, Moore M, et al. Implementing interventions to reduce antibiotic use: a qualitative study in high-prescribing practices. BMC Family Practice. 2021 Jan 23;22(1):25.

4. Cane J, O'Connor D, Michie S. Validation of the theoretical domains framework for use in behaviour change and implementation research. Implementation Science. 2012;7(1):37.

5. Michie S, Richardson M, Johnston M, Abraham C, Francis J, Hardeman W, et al. The behavior change technique taxonomy (v1) of 93 hierarchically clustered techniques: building an international consensus for the reporting of behavior change interventions. Annals of Behavioral Medicine. 2013 Aug;46(1):81–95.

6. Little P, Stuart B, Francis N, Douglas E, Tonkin-Crine S, Anthierens S, et al. Effects of internet-based training on antibiotic prescribing rates for acute respiratory-tract infections: a multinational, cluster, randomised, factorial, controlled trial. The Lancet. 2013 Oct 5;382(9899):1175–82.

7. Francis NA, Butler CC, Hood K, Simpson S, Wood F, Nuttall J. Effect of using an interactive booklet about childhood respiratory tract infections in primary care consultations on reconsulting and antibiotic prescribing: a cluster randomised controlled trial. BMJ. 2009 Jul 29;339:b2885.

8. Gulliford MC, Prevost AT, Charlton J, Juszczyk D, Soames J, McDermott L, et al. Effectiveness and safety of electronically delivered prescribing feedback and decision support on antibiotic use for respiratory illness in primary care: REDUCE cluster randomised trial. BMJ. 2019 Feb 13;364:l236.

9. Hoffmann TC, Glasziou PP, Boutron I, Milne R, Perera R, Moher D, et al. Better reporting of interventions: template for intervention description and replication (TIDieR) checklist and guide. BMJ. 2014 Mar 7;348:g1687–g1687.

10. Duncan E, O'Cathain A, Rousseau N, Croot L, Sworn K, Turner KM, et al. Guidance for reporting intervention development studies in health research (GUIDED): an evidence-based consensus study. BMJ Open. 2020 Apr 1;10(4):e033516.
